# Combined Anterior Cruciate Ligament and Anterolateral Ligament Reconstruction Using a Single Achilles Tendon Allograft: A Technical Note

**DOI:** 10.3390/medicina58070929

**Published:** 2022-07-13

**Authors:** Chul-Soo Lee, Seung-Beom Han, Ki-Mo Jang

**Affiliations:** Department of Orthopaedic Surgery, Anam Hospital, Korea University College of Medicine, Seoul 02841, Korea; anotherlife@naver.com (C.-S.L.); sbhan1107@gmail.com (S.-B.H.)

**Keywords:** knee joint, ALL reconstruction, ACL reconstruction, rotational instability, technique

## Abstract

Clinical outcomes after anterior cruciate ligament reconstruction (ACLR) have improved remarkably. However, residual rotational instability of the knee joint remains a major concern. The anterolateral ligament (ALL) has recently gained interest as a secondary stabilizer of knee joint rotatory instability, and this has led to the attempt of ALL reconstruction (ALLR) in combination with ACLR to restore rotational stability in patients with anterior cruciate ligament (ACL) injury. Although several techniques for ALLR have recently been introduced, the ideal graft type and surgical technique for combined ACLR and ALLR are yet to be established. This technical note therefore aimed at introducing a novel surgical procedure involving the use of a single Achilles tendon allograft as a relatively simple and minimally invasive procedure for combined ALL and ACL reconstruction.

## 1. Introduction

Clinical outcomes after anterior cruciate ligament reconstruction (ACLR) have remarkably improved due to the accumulating knowledge of the anatomy and biomechanics of the anterior cruciate ligament (ACL) and advances in surgical techniques [1,2]. However, some patients continue to complain of residual rotational instability and have difficulties returning to their pre-injury level of sports activities after isolated ACLR [3,4,5,6]. To address this issue, anterolateral ligament reconstruction (ALLR) has recently attracted interest as a potential solution [7].

The anterolateral ligament (ALL) is an extra-articular ligamentous structure located on the anterolateral aspect of the knee joint. Since the re-discovery of the ALL by Claes et al. [8], there have been numerous studies regarding its anatomy and biomechanics. Although there still exist controversies regarding its anatomy, it is known to originate proximal and posterior to the lateral femoral epicondyle (LFE), to course antero-distally, and to insert broadly at approximately 5–10 mm below the lateral tibial plateau, midway between Gerdy’s tubercle and the head of the fibula [9,10]. Recent studies have shown that the ALL functions as a secondary stabilizer to the ACL in resisting anterior tibial translation and internal tibial rotation [11,12,13]. Several biomechanical studies have reported that the combination of extra-articular ALL reconstruction (ALLR) and ACLR yielded better results than isolated intra-articular ACLR, especially with respect to post-operative knee joint stability [14,15,16,17]. Furthermore, a recent meta-analysis demonstrated that combined ACL and ALL reconstruction had better post-operative clinical outcomes than that of isolated ACLR [7].

Several surgical techniques have recently been suggested for combined ALL and ACL reconstruction. Helito et al. recommended the use of a tripled semitendinosus auto- or allograft in conjunction with a gracilis auto- or allograft for a combined ACL and ALL reconstruction [18]. Sonnery-Cottet et al. used a doubled gracilis tendon graft for ALLR that was placed in an inverted V-shape pattern with two points of fixation on the tibia, in an attempt to mimic the broad-based tibial attachment of the native ALL [19]. Smith et al. described an all-inside quadrupled semitendinosus ACLR through a minimally invasive approach, using a single gracilis graft for ALLR [20]. However, the ideal graft to use and surgical technique for combined ACL and ALL reconstruction have not yet been clearly established.

The purpose of this technical note is to introduce a novel surgical procedure using a single Achilles tendon allograft as a relatively simple and minimally invasive procedure for combined ALL and ACL reconstruction.

## 2. Surgical Technique

### 2.1. Patient Setup

The patient is anesthetized (general or spinal anesthesia) and positioned supine in the standard arthroscopy position with a lateral post proximal to the knee. The knee joint is positioned slightly past the distal break point of the operating table to place the limb in a 90° flexion position. As such, the knee can freely undergo its full range of motion. A thigh tourniquet is applied and maintained inflated throughout the procedure. Physical examination, including the Lachman and pivot shift tests, are performed to evaluate anteroposterior and rotatory instability.

### 2.2. Arthroscopic Examination and Tibial and Femoral Tunnel Preparation for ACLR

**Figure 1:**
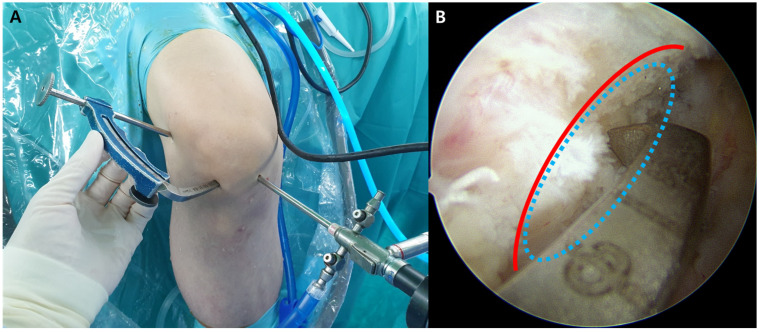
Creation of an anatomical femoral tunnel using the outside-in technique. (**A**) After palpation of the lateral femoral epicondyle, a small incision is made just proximal and posterior to the lateral femoral epicondyle. The guide’s angulation is adjusted to allow the tip of drill sleeve placement in the incision at the femoral footprint of the anterolateral ligament. (**B**) The tip of the femoral aiming guide is placed at the femoral footprint of the anterior cruciate ligament through the anterolateral portal under direct arthroscopic visualization through the anteromedial portal (red line, lateral intercondylar ridge (Resident’s ridge)); blue ellipse, femoral footprint of the anterior cruciate ligament).

After setting up the patient, routine arthroscopic examination is performed through the standard anterolateral and anteromedial portals. After complete intra-articular examination, any concomitant pathology of the menisci or articular cartilage is managed prior to ACLR. Subsequently, a femoral tunnel is created for both the ACLR and ALLR using the ACL RetroConstruction System(Arthrex, Naples, FL, USA) and FlipCutter drill (Arthrex, Naples, FL, USA) through the anatomic outside-in retrograde-reaming technique. The tip of the femoral aiming guide is placed through the anterolateral portal at the ACL femoral footprint under direct arthroscopic visualization through the anteromedial portal. After palpation of the LFE, a 15 mm incision is made just proximal and posterior to the LFE. The guide’s angulation is adjusted to allow the tip of drill sleeve placement in the incision at the femoral footprint of the ALL (approximately 5 mm proximal and posterior to the LFE) (Figure 1). Fluoroscopy or radiography can help ensure the exact footprint positions of the ALL. The femoral tunnel (9–10 mm in diameter) is then created such that the outlet of the tunnel is approximately 5 mm proximal and posterior to the LFE, corresponding to the femoral footprint of the ALL [8]. As such, the inlet of the femoral tunnel would be located at the femoral footprint of the ACL, and its outlet would be located at the femoral footprint of the ALL. After this, the ACL tibial tunnel (9–10 mm in diameter) is created in an outside–inside fashion using an angled tibial guide. A 55°-angled ACL tibial guide is inserted into the knee joint through the anteromedial portal. The tip of the ACL tibial guide is positioned in the center of the ACL tibial footprint. A guide pin is inserted from the anteromedial aspect of the proximal tibia. If the pin has exited exactly in the center of the ACL tibial footprint, the tibial tunnel is created using a reamer with the same size as the femoral tunnel diameter. A suture loop is created by passing an Ethibond suture (Ethicon, Edinburgh, UK) in a retrograde fashion from the femoral tunnel to the tibial tunnel; this later serves as the graft passage.

### 2.3. Preparation of Two Tibial Bony Sockets for ALLR

A small transverse skin incision is made on the tibial footprint of the ALL, which is located midway between Gerdy’s tubercle and the tip of the fibular head (approximately 20 mm posterior to the center of Gerdy’s tubercle), approximately 10 mm below the lateral joint line [10]. Two tibial bony sockets are created for the fixation of the two bundles of the ALL graft: one socket is located halfway between Gerdy’s tubercle and the tip of the fibular head (at approximately 5 to 10 mm below the lateral joint line), and the other socket is located approximately 10 mm anteriorly from the first one toward Gerdy’s tubercle. The two bony sockets (20 mm depth) are created parallelly using a 4.5 mm reamer following a guide pin. Subsequently, each bundle of the ALL graft is fixed into each tibial socket using a 4.75 mm SwiveLock tenodesis screw (Arthrex, Naples, FL, USA).

### 2.4. Graft Preparation

**Figure 2:**
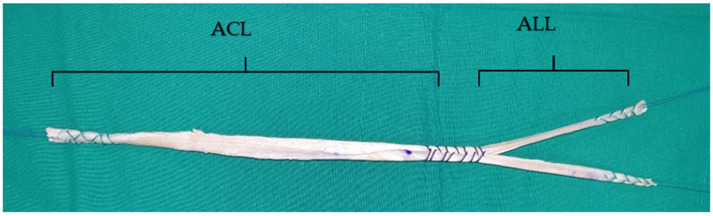
Graft preparation for combined ACL and ALL reconstruction using a single Achilles tendon allograft. The allograft is trimmed into a Y-shape graft with two portions: an ACL portion and a two-bundle ALL portion.

After creating the bony tunnels and sockets for ACLR and ALLR, the footprint distances and tunnel lengths are measured to determine the appropriate length of the tendon graft. The Achilles tendon allograft is trimmed into a Y-shaped graft consisting of two portions: an ACL portion (9–10 mm in diameter) and an ALL portion with two bundles (5 mm in diameter each). The end of the ACL portion is sutured using the whip-stitch method, with an Ethibond suture (Ethicon, Edinburgh, UK). The ALL portion is divided into two bundles (with each end sutured in the same manner) for later fixation into the two ALL tibial sockets. At the bifurcation site from the ACL portion to the ALL portion, an additional whip-stitched suture is made, approximately 30 mm in length, in the direction of the ACL portion, to prevent possible tendon splitting (Figure 2).

### 2.5. Graft Passage and Fixation for the Combined ACL and ALL Reconstruction

**Figure 3:**
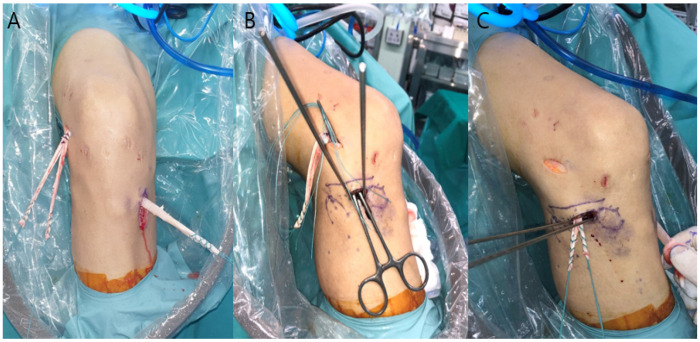
(**A**) ACL graft positioned in the femoral and tibial tunnels, and ALL graft pulled outside the femoral tunnel outlet. (**B**,**C**) Clamp passed deep into the iliotibial tract to drive the ALL graft into the tibial bony sockets.

**Figure 4:**
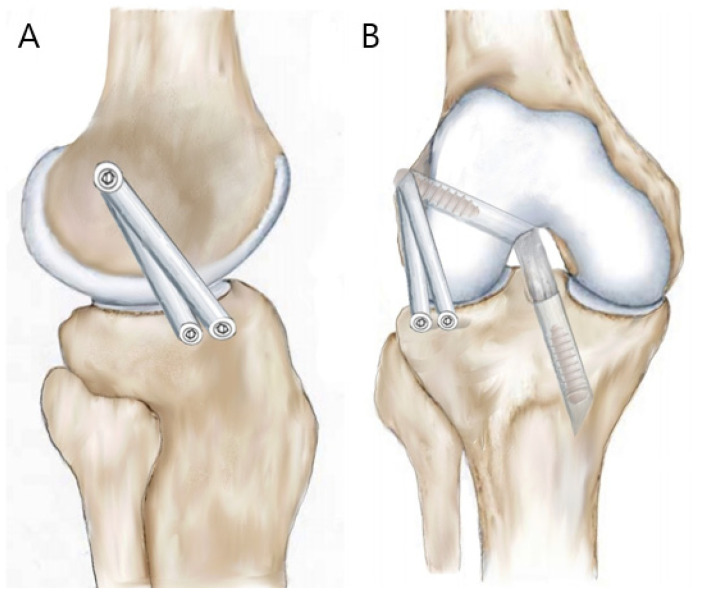
Schematic illustration of combined ACL and ALL reconstruction using a single Achilles tendon allograft shown in lateral (**A**) and frontal views (**B**).

The prepared Achilles tendon allograft is attached to the loop outside the tibial tunnel and then pulled through the tibial and femoral tunnels passing over the joint. The two bundles of the ALL graft are pulled and positioned outside the femoral tunnel outlet. The graft is then sequentially fixed for ACL and ALL reconstructions. First, the bifurcated transitional portion of the graft is fixed at the femoral tunnel using a bioabsorbable interference screw (Arthrex, Naples, FL, USA). For ACLR, after graft pre-tensioning, the distal portion of the ACL graft is fixed at the tibial tunnel (with the knee in full extension and neutral position) using a bioabsorbable interference screw (Arthrex, Naples, FL, USA) followed by a post-tie fixation. Subsequently, for the ALLR, the two bundles of the ALL graft from the femoral tunnel outlet are directed deep to the iliotibial band (ITB) percutaneously and delivered through a distal skin incision to the tibial sockets (Figure 3). The knee is then moved through its different ranges of motion to ensure non-isometry of the ALL graft, which is normally tense in extension and slack in flexion. Each bundle of the ALL graft is pulled while appropriate tension is maintained on the graft with the knee at 30° flexion and in a neutral position. The individual bundles are then fixed into the respective prepared tibial sockets using a 4.75 mm SwiveLock Tenodesis screw (Arthrex, Naples, FL, USA). After the fixation, the excess portion of the graft is cut (Figure 4).

## 3. Discussion

As a potential solution for residual rotatory instability after isolated ACLR, combined ACL and ALL reconstruction has recently gained interest among orthopedic knee specialists. Different surgical techniques for combining ACLR and ALLR have already been reported to have favorable clinical outcomes [18,19,20]. The aforementioned novel technique of combined ACL and ALL reconstruction uses a single Achilles tendon allograft. Through this technique, anatomical ACL and ALL reconstructions are performed by sharing a single femoral tunnel. For tibial fixation during ALL reconstruction, two tibial bony sockets are created to mimic the relatively broad tibial footprint of the native ALL. This novel technique may be more useful for revision procedures wherein a remnant autograft is not available. However, it can also be applied to primary cases.

The proposed technique offers several advantages. First, by trimming a single Achilles tendon allograft into two parts consisting of the ALL and ACL portions, there is no need to use additional graft tissue. As the Achilles tendon allograft is sufficient in width and length, it is possible to produce a single graft sufficient to realize the combined ACL and ALL reconstruction by performing graft preparation as described above. This may also be beneficial to the patient as it may help reduce the cost of the surgery. Second, by performing the combined ALLR and ACLR through a common single femoral tunnel, the potential risk of overlap between the two femoral tunnels (in case tunnels for the ALLR and ACLR procedures are made separately) can be avoided. Furthermore, sharing a common femoral tunnel for the fixation of the ALL and ACL graft could contribute to a decrease in the operative time, thus preventing possible intra- and post-operative complications. Third, through a minimally invasive approach, this technique can minimize complications such as ITB injuries. Fourth, as a technique using only a single allograft tendon, donor site morbidity such as weakness of the hamstring muscle and anterior knee pain, caused by the harvesting of the autografts, can be avoided. Additionally, this technique has the advantage of being easy to apply in cases of revisional reconstructions, where the use of autografts is difficult. Finally, although some studies reported ALL reconstruction techniques using a single bundle graft [20,21], the ALL is reconstructed using two tibial bony sockets with two graft bundles in this technique [22,23,24]. The two tibial sockets in this novel technique may have the advantage of restoring the broad base of the anatomic ALL tibial footprint. Previous anatomical studies of the ALL have demonstrated that the native ALL has a broad-based tibial insertion [10,25]. Furthermore, a separate tibial fixation using the two graft bundles fixed in different directions (in the shape of an inverted V) can function complementarily in the maintenance of graft tension and length during knee motion and mimic the anatomical characteristics of the native ALL.

The potential risks of this technique include irritation of the iliotibial band (by the protruding interference screws), anterolateral tibial plateau fracture, and tibial tunnel collision during the creation of the bony tunnels. To avoid these possible complications, it is crucial to insert the screws such that their ends do not protrude outside the cortex when fixing the interference screw in the lateral femoral tunnel. It is also recommended to maintain the two tibial sockets at a distance of approximately 10 mm from each other (so that the two sockets do not overlap), as well as to consider the direction of the sockets so as to prevent tibial tunnel collision or tibial plateau fracture. Lastly, this novel procedure uses an Achilles tendon allograft. Graft selection is one of the important issues in ACLRs. Although ACLRs using autografts have consistently yielded good clinical outcomes, the incidence of post-operative donor site morbidity and the desire to avoid autologous tissue sacrifice have prompted a consideration of allografts [26,27,28]. Although allografts have some advantages such as a shorter operation, less pain during initial recovery, and no donor site morbidity, there are some disadvantages including slower graft incorporation, increased cost, and concerns about disease transmission and higher rupture rate in young active patients [28,29]. Although there are still controversies regarding graft selection in ACLRs, some recent studies have reported that clinical outcomes with autografts are as good as or slightly better than those with allografts [29,30,31].

## 4. Conclusions

Although several techniques for ALLR have recently been introduced, the ideal graft type and surgical technique for combined ACL and ALL reconstruction are yet to be established. To the best of our knowledge, this is the first surgical technique that combines ACLR and ALLR using a single Achilles tendon allograft. This relatively facile and minimally invasive procedure will be beneficial in restoring the function of the native ACL and ALL in patients who present with both anteroposterior and anterolateral rotatory knee instabilities. Further biomechanical and long-term clinical studies are necessary to validate this novel technique for combined ACL and ALL reconstruction by using a single Achilles tendon allograft.

## Data Availability

Not applicable.
